# Time trend analysis of gastric cancer incidence in Japan by histological types, 1975-1989

**DOI:** 10.1054/bjoc.2000.1602

**Published:** 2001-02

**Authors:** S Kaneko, T Yoshimura

**Affiliations:** Department of Clinical Epidemiology, Institute of Industrial Ecological Sciences, University of Occupational and Environmental Health, Japan

**Keywords:** gastric cancer, histological type, time trend, epidemiology

## Abstract

Since different histological types (HT) of gastric cancer (GC) may differ in their aetiology, time trend analysis by HT may afford an insight into aetiology. From the Gastric Cancer Registry of Japan, 161 067 cases diagnosed were retrieved between 1975 and 1989 to calculate the annual relative frequencies, stratified by age group and sex, of HT according to the Lauren and the Japanese Research Society for Gastric Cancer (JRSGC) classifications. Age- and sex-specific incidence rates by HT were estimated by multiplying the corresponding national cancer incidence rates of GC by the relative frequencies. Logistic regression models stratified by sex and age group were fitted to determine the time trends of HT. Using the Lauren classification, a decreasing trend of the intestinal type and a stable trend of the diffuse type were found. By the JRSGC classification, significant decreasing trends for most age groups were found for papillary and mucinous adenocarcinomas. Tubular adenocarcinomas (well differentiated type) showed a decreasing trend only in younger age groups. Tubular (moderately differentiated type), poorly differentiated adenocarcinomas, and signet ring cell carcinoma were statistically stable during the period. Considering changes in lifestyles of the Japanese, the result suggests that there are three aetiological types of GC. © 2001 Cancer Research Campaign http://www.bjcancer.com


*British Journal of Cancer* (2001) **84**(3), 400–405
© 2001 Cancer Research Campaign
doi: 10.1054/ bjoc.2000.1602, available online at http://www.idealibrary.com on IDEAL®

http://www.bjcancer.com

### Time trend analysis of gastric cancer incidence in Japan by histological types, 1975–1989

**S Kaneko and T Yoshimura**

Department of Clinical Epidemiology, Institute of Industrial Ecological Sciences, University of Occupational and Environmental Health, Japan

**Summary** Since different histological types (HT) of gastric cancer (GC) may differ in their aetiology, time trend analysis by HT may afford an insight into aetiology. From the Gastric Cancer Registry of Japan, 161 067 cases diagnosed were retrieved between 1975 and 1989 to calculate the annual relative frequencies, stratified by age group and sex, of HT according to the Lauren and the Japanese Research Society for Gastric Cancer (JRSGC) classifications. Age- and sex-specific incidence rates by HT were estimated by multiplying the corresponding national cancer incidence rates of GC by the relative frequencies. Logistic regression models stratified by sex and age group were fitted to determine the time trends of HT. Using the Lauren classification, a decreasing trend of the intestinal type and a stable trend of the diffuse type were found. By the JRSGC classification, significant decreasing trends for most age groups were found for papillary and mucinous adenocarcinomas. Tubular adenocarcinomas (well differentiated type) showed a decreasing trend only in younger age groups. Tubular (moderately differentiated type), poorly differentiated adenocarcinomas, and signet ring cell carcinoma were statistically stable during the period. Considering changes in lifestyles of the Japanese, the result suggests that there are three aetiological types of GC. © 2001 Cancer Research Campaign http://www.bjcancer.com

**Keywords**: gastric cancer; histological type; time trend; epidemiology

Gastric cancer, though still one of the major causes of death among the Japanese, is declining (Tominaga, 1987). Since certain reported risk factors of gastric cancer have also declined over the last three or four decades in Japan, e.g. improved standards, changing of dietary habits, wider use of refrigerators (Hirayama, 1984), and a decreasing rate of *Helicobacter pylori (H. pylori)* infection (Asaka et al, 1992; Haruma et al, 1997), a continued decline in the incidence of gastric cancer is expected.

Several studies have reported that of two histological types of gastric cancer (Lauren, 1965) the intestinal type is more susceptible to the environmental factors: and a diffuse type less susceptible to such factors (Munoz and Connelly, 1971; Howson et al, 1986; Munoz et al, 1968; Parsonnet et al, 1991b).

In this study, we have attempted to estimate incidence rates of gastric cancer by histological type and analyse their time trends.

### MATERIAL AND METHODS

### The Gastric Cancer Registry of Japan

The Gastric Cancer Registry of Japan was founded in 1969 to investigate the diagnosis, treatment and prognosis of this cancer among patients diagnosed since 1963 and admitted to participating hospitals whether or not for surgical treatment (National Cancer Center, 1972). The Registry was co-ordinated by the National Cancer Center, the Japanese Research Society for Gastric Cancer (JRSGC), and Miwa Registry Institute for Stomach Cancer (MRISC); it closed in 1990. A wide variety of hospitals with surgical units participated.

*Received 26 June 2000*
*Revised 6 October 2000*
*Accepted 30 October 2000*

*Correspondence to:* S Kaneko

In 1989, 13 033 cases were registered from 188 institutions from all over Japan. The registration and follow up forms collected details of address, sex, occupation, family history, past history, age at first diagnosis, diagnostic methods and date, TMN stage, treatment, histopathological diagnosis, and survival status of each patient provided by the periodical follow-up survey inquired to hospitals. The histology was classified according to the JRSGC classification of gastric cancer, which has been broadly accepted and used among Japanese oncologists in clinical practice (JRSGC, 1995). Since the registration form was revised according to major revision of the JRSGC classification in 1974, cases registered since 1975 have been used in this study.

### Data retrieval from the Registry and incidence rate estimation of gastric cancer by histological types

From 178 252 registered cases at ages 30 and over at first diagnosis from 1975 to 1989, we retrieved 161 067 cases with complete data on sex, birth date, histological diagnosis and date of diagnosis of which 157 558 were surgically and 3509 were non-surgically treated. We excluded 1997 cases because they were coded to other sites according to the International Classification of Diseases (the ninth version) (e.g., malignant lymphoma) and 15 188 cases without a histological diagnosis. After retrieval, the JRSGC classification was converted to that by Lauren (Hanai and Fujimoto, 1982) (Table 1).

Annual proportional frequencies of histological types of gastric cancer were computed for each sex and 5-year age group at first diagnosis using both classifications. Annual incidence rates of gastric cancer by histological types were calculated by multiplying the national incidence rates of total gastric cancer (RGPCR, 1999a) by the proportional frequencies of each histological type in each age group and sex in the relevant year. Annual age standardized rates (ASRs) by histological types were calculated using a 'Model' population for 1985 in Japan as the standard population (Abe et al, 1991).

### Statistical method for time trend analysis

By fitting a logistic regression model, age group stratified time trends were analysed with disease status as a dependent variable and time (year) as an independent variable using 5-year-age-group stratified data (12 age groups from 30–34 age group to over-85 age group) as follows:

$$\ln(E(n/N)) = \alpha + \beta(Year)$$

where $n$ denotes estimated number of cases in a sex-age group in a year, $N$ denotes the general population in that group in the same year, and *year* denotes a categorical variable from 1 to 15 for the years between 1975 and 1989.

**Table 1** Converting table[a] from the JRSGC to the Lauren classification

| Lauren Classification | JRGSC Classification |
|---|---|
| Intestinal type | Papillary adenocarcinoma<br>Tubular adenocarcinoma<br>Well differentiated type<br>Moderately differentiated type<br>Mucinous adenocarcinoma |
| Diffuse type | Signet ring cell carcinoma<br>Poorly differentiated adenocarcinoma<br>Undifferentiated carcinoma[b] |
| Others | Adenoacanthoma<br>Squamous cell carcinoma<br>Miscellaneous (unclassified) carcinoma<br>Carcinoid tumour |

[a]This table was modified from Hanai's converting table (Hanai and Fujimoto, 1982) regarding histological types of the revised JRSGC classification in 1974.
[b]A histological type of undifferentiated carcinoma was classified into others in the JRSGC classification in this study, since the number of diagnoses was quite small.

Percentages of average increase or decrease per year were computed with 95% confidence intervals. The results for linear trend analysis were presented by percentage of increase in incidence rate per year (% increase). STATA statistical package (version 6.0) was used with the glogit procedure to fit a logistic regression model for grouped data (STATA Corp., 1999).

### RESULTS

The estimated annual cover rates for gastric cancer in Japan between 1975 and 1989 are shown in Table 2. The cover rates in most age groups showed 10–20% coverage by the Registry. The cover rates within the same age group were relatively stable during the observation period.

### Time trend using the Lauren classification

Temporal changes in relative frequencies of histological types by the Lauren classification for all age groups are shown in Figure 1, along with those for the JRSGC classification. These frequencies were relatively stable; and diffuse type between 1975 and 1989: approximately 62–58% were intestinal and 34–40% diffuse among males, and 40–46% and 49–58% respectively among females.

Changes in the relative frequencies of Lauren histological types in the period 1975–1989 are shown by age group in Figure 2. The diffuse type was the major histological type at younger ages, while the intestinal type became predominant among the elder age groups (Figure 2).

Age standardized rates (ASRs) of total gastric cancer and the two Lauren types for the over 30-year-old population are shown in Figure 3 on a log scale. Total gastric cancer and the intestinal type showed a decreasing trend for males and females. On the other hand, the diffuse type was relatively stable. Results of linear trend analyses for total gastric cancer and the two Lauren types by age group and sex with 95% confidence intervals are shown in Figure 5. The incidence rates of total gastric cancer showed a significant decreasing trend in younger age groups for males and in most age groups for females. The incidence rate for the over-85 age group, however, showed increasing trends both for males and females. In

**Table 2** Estimated cover rates[a] (%) of gastric cancer patients throughout Japan by the Gastric Cancer Registry of Japan, 1975–1989

| Registration year | Age group 30–34 | 35–39 | 40–44 | 45–49 | 50–54 | 55–59 | 60–64 | 65–69 | 70–74 | 75–79 | 80–84 | 85 |
|---|---|---|---|---|---|---|---|---|---|---|---|---|
| 1975 | 12.6 | 26.1 | 14.9 | 15.0 | 13.3 | 11.5 | 10.9 | 9.5 | 6.4 | 1.6 | 5.1 | 0.7 |
| 1976 | 17.9 | 33.4 | 15.5 | 15.4 | 13.3 | 15.1 | 12.7 | 10.8 | 7.9 | 1.9 | 6.3 | 0.6 |
| 1977 | 15.5 | 27.2 | 13.3 | 14.2 | 13.8 | 14.4 | 11.2 | 10.1 | 8.1 | 2.1 | 6.2 | 1.1 |
| 1978 | 16.2 | 26.5 | 35.7 | 15.8 | 14.9 | 16.1 | 13.6 | 11.6 | 9.7 | 2.5 | 7.6 | 1.2 |
| 1979 | 17.4 | 31.2 | 17.4 | 17.8 | 15.2 | 15.5 | 13.8 | 12.9 | 10.0 | 3.0 | 9.3 | 1.1 |
| 1980 | 16.8 | 30.5 | 20.0 | 17.8 | 17.2 | 16.1 | 14.8 | 13.0 | 10.2 | 3.5 | 8.9 | 1.4 |
| 1981 | 19.9 | 31.0 | 18.7 | 17.7 | 18.5 | 16.6 | 14.8 | 13.5 | 10.9 | 3.9 | 9.8 | 1.4 |
| 1982 | 17.2 | 29.2 | 18.8 | 17.9 | 16.8 | 15.9 | 15.7 | 13.6 | 11.7 | 4.2 | 9.7 | 0.9 |
| 1983 | 18.5 | 29.5 | 18.2 | 18.8 | 16.0 | 16.5 | 15.7 | 14.5 | 12.2 | 4.7 | 9.8 | 1.5 |
| 1984 | 17.2 | 39.8 | 17.9 | 18.8 | 16.3 | 15.9 | 15.6 | 14.7 | 11.5 | 5.0 | 10.1 | 2.5 |
| 1985 | 18.5 | 38.9 | 17.9 | 19.1 | 16.6 | 16.0 | 15.6 | 16.1 | 12.9 | 5.8 | 11.4 | 1.9 |
| 1986 | 18.9 | 50.6 | 22.3 | 20.3 | 20.2 | 18.6 | 17.5 | 16.6 | 14.6 | 6.4 | 13.7 | 2.6 |
| 1987 | 18.9 | 59.5 | 20.0 | 19.9 | 19.2 | 18.7 | 17.7 | 16.9 | 14.8 | 6.6 | 14.7 | 2.3 |
| 1988 | 15.9 | 53.0 | 18.2 | 16.5 | 16.9 | 17.3 | 15.5 | 15.4 | 13.1 | 6.1 | 13.6 | 2.6 |
| 1989 | 14.5 | 46.3 | 16.1 | 16.0 | 15.4 | 15.2 | 14.5 | 13.9 | 12.0 | 5.6 | 11.3 | 2.3 |

[a]Cover rates were calculated by dividing registered cases in the Registry by incidences of gastric cancer in Japan estimated from several population-based cancer registries (RGPCR, 1999b).

© 2001 Cancer Research Campaign 

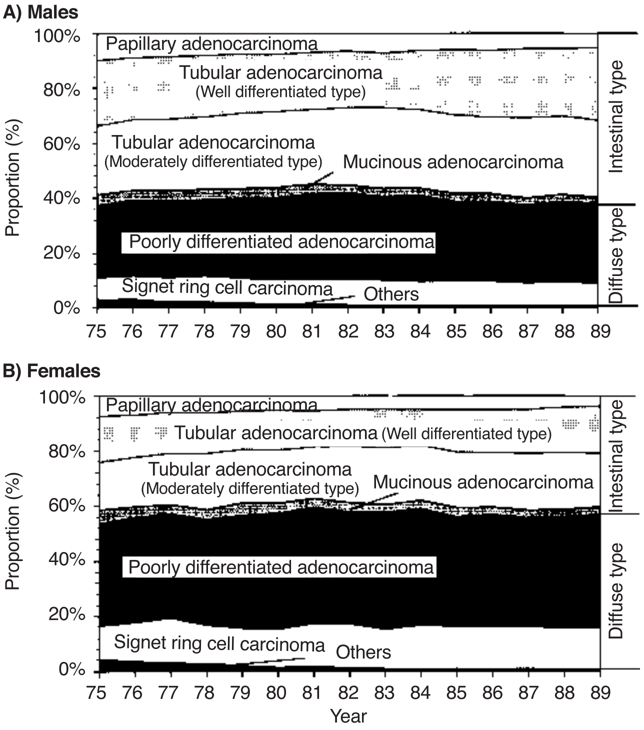

**Figure 1** Temporal transition in relative frequencies of histological types by the JRSGC classification for all age groups, 1975–1989

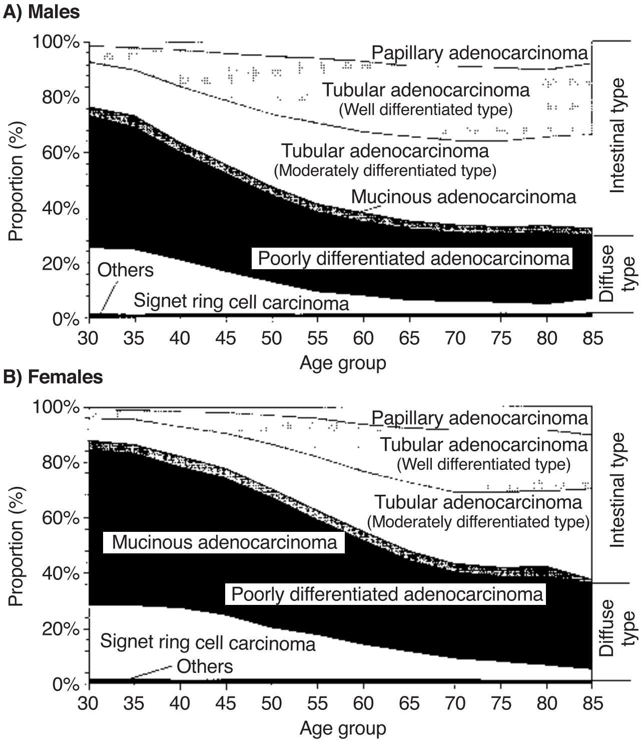

**Figure 2** Age transition of relative frequency of histological types of gastric cancer during the whole period of 1975–1990 in the Gastric Cancer Registry of Japan

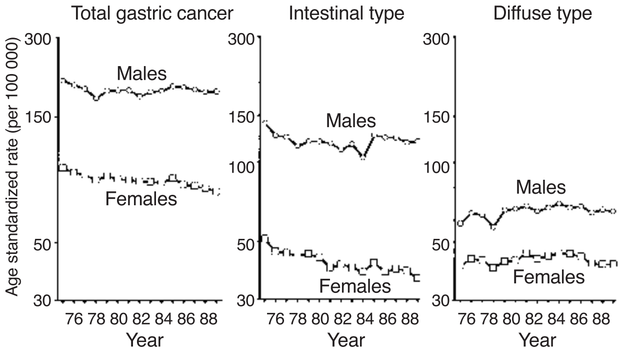

**Figure 3** Time trend estimation of age standardized incidence rates (ASRs) for total gastric cancer and histological types of the Lauren for over 30-year-old groups from 1975 to 1989 (log scale)

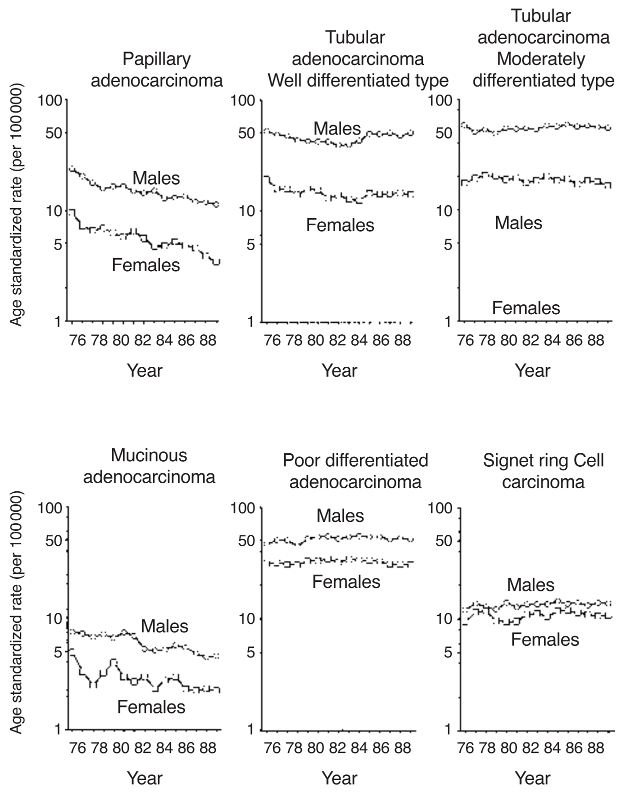

**Figure 4** Time trend estimation of age standardized incidence rates (ASRs) for gastric cancer by histological types of the JRSGC for over 30-year-old groups from 1975 to 1989 (log scale)

the intestinal type, decreasing trends were observed in younger age groups for males, and most age groups for females. The trends of the diffuse type were relatively stable in most age groups both for males and females (Figure 5).

### Time trend using the JRSGC classification

Temporal changes in relative frequencies of histological types by the JRSGC classification for all age groups are also shown in Figure 1. The relative frequencies of papillary adenocarcinoma in 1989 were reduced by about 50% of those in 1975 for both males and females. Other histological types showed slight increases over the study period. Corresponding changes by age group and sex are shown in Figure 2. Poorly differentiated adenocarcinoma was the major type among the younger age groups, while tubular

© 2001 Cancer Research Campaign

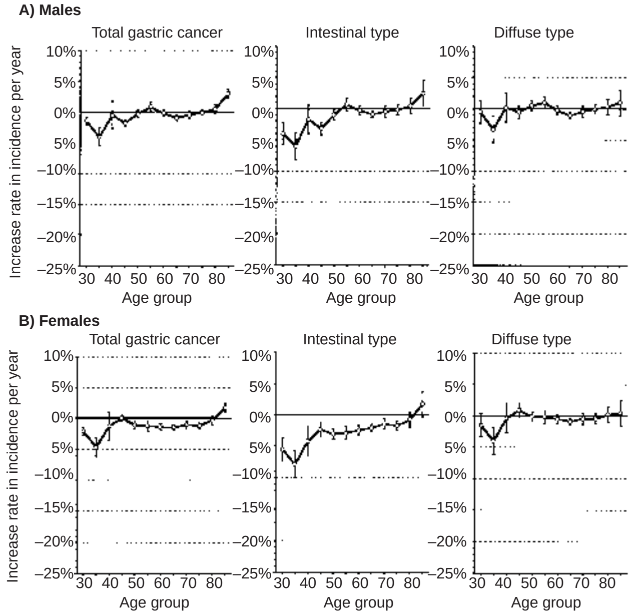

**Figure 5** Sex-age-group stratified linear trends for total gastric cancer and histological types of the Lauren for over 30-year-old groups by logistic regression model with 95% confidence intervals in males (**A**) and females (**B**)

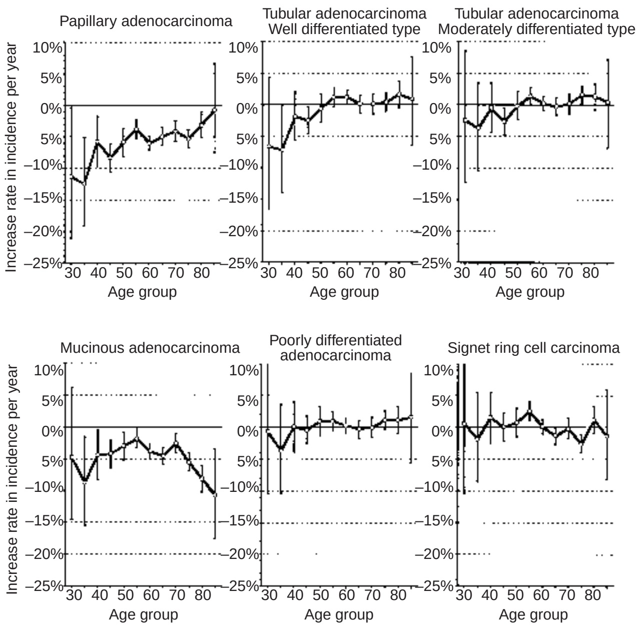

**Figure 6** Age-group stratified linear trend analyses for gastric cancer by histological types of the JRSGC classification over 30-year-old groups by logistic regression model with 95% confidence intervals in males adenocarcinoma (moderately differentiated type) became dominant at older ages among both males and females (Figure 2). The ASR trends by histological types in the JRSGC classification are shown in Figure 4 on a log scale. In both sexes, a decreasing incidence was seen for papillary and mucinous adenocarcinomas, and possibly tubular adenocarcinoma (well differentiated). Other JRSGC types were stable over the study period. The declining trends for papillary and mucinous adenocarcinomas applied to most age groups both in males and females. Tubular adenocarcinoma (moderately

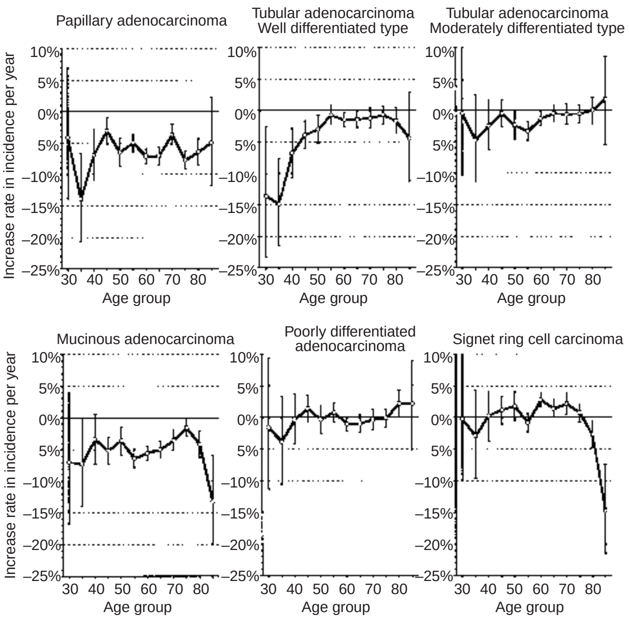

**Figure 7** Age-group stratified linear trend analyses for gastric cancer by histological types of the JRSGC classification over 30-year-old groups by logistic regression model with 95% confidence intervals in females differentiated type), poorly differentiated adenocarcinoma, and signet ring cell carcinoma showed statistically stable trends in almost all age groups in both sexes. Tubular adenocarcinoma (well differentiated type) showed a significant declining trend only in younger males and females (Figures 6 and 7).

### DISCUSSION

Several studies have suggested that the decline in the incidence of gastric cancer is due to a decrease of the Lauren intestinal-type (Munoz and Connelly, 1971; Amorosi et al, 1988; Nikulasson et al, 1992; Lauren and Nevalainen, 1993). However, these studies were mostly based on the proportional frequency ratio of the intestinal and diffuse types which does not itself indicate trends of incidence.

Age-adjusted incidence rates (ASR) of the intestinal type showed a decreasing trend and a stable trend in the diffuse type. Although these results are in good agreement with past studies (Munoz et al, 1968; Munoz and Connelly, 1971; Hansson et al, 1991), age group stratified time trend analyses might be more informative if exposure status to the relevant environmental factors differed between age groups.

The age group stratified time trends of the intestinal type were clearly decreased in younger age groups both for males and females, and stable for males or slightly decreased for females in elder age groups. In the time trends of the JRSGC classification corresponding to the intestinal type, three patterns of time trends could be identified; 1) a pattern showing linear decreasing trends in most age groups, 2) a pattern showing linear decreasing trends only in younger age groups, and 3) a pattern of relatively stable trends in most age groups during the observation period.

The first pattern could reflect an evenly decreasing chance of exposure to the environmental risk factors of gastric cancer for all

*© 2001 Cancer Research Campaign*

age groups. Since food/nutrient intake among the Japanese has moved towards protein and fat rich food (Tominaga and Kuroishi, 1997), these factors might work protectively against these histological types of gastric cancer for all age groups.

The second pattern might be due to different exposures at younger and older ages. The *H. pylori* infection rate is reported to be decreasing in young age groups, but still high among the elderly population in Japan (Asaka et al, 1992; Haruma et al, 1997) so this could produce such a trend pattern. In addition, differences in past and current lifestyles between the younger and elder age groups might also contribute to the trend, e.g. differences in hygiene status in childhood and differences in preference of current food intakes between the age groups.

The third trend pattern might be independent of environmental factors attributed to lifestyles and be genetic in nature; this may be relevant to the diffuse type.

Some studies have concluded that *H. pylori* infection has an association not only with the intestinal type, but also the diffuse type (Nomura et al, 1991; Parsonnet et al, 1991a; Sipponen et al, 1992; Asaka et al, 1994); however, the association has not been definitively identified (Talley et al, 1991). A meta-analysis of 42 case-control studies on *H. pylori* and gastric cancer revealed that the intestinal type had more associated with *H. pylori* (Eslick et al, 1999). If we suppose that a histological type of gastric cancer has an association with *H. pylori*, its time trend would be similar to the second trend pattern in this paper, that is, a decreasing trend only in younger age group where a prevalence of *H. pylori* has been declining. However, such a pattern was not observed in the diffuse type, and even in some JRSGC histological types corresponding to the intestinal type. The stable gastric cancer trends regardless of changes in *H. pylori* infection rate may give us a clue to elucidate the relationship between gastric cancer and *H. pylori* along with a phenomenon that low frequency of gastric cancer in several developing countries where the prevalence of *H. pylori* infection is as high as 90% (Crespi and Citarda, 1998).

The possibility that cases in the Registry are not representative of gastric cancer in Japan could be a limitation of this study. Since reporting biases with respect to histological type from hospitals and selective visits or referrals of gastric cancer cases to participating hospitals are considered to be small, the relative frequencies of histological types of gastric cancer in the registry should be similar to those of all cases in Japan.

Another limitation is the possibility of changes in histopathological diagnosis. In Japan, the JRSGC classification has been broadly accepted by oncologists and since this has not been drastically revised since 1974 (JRSGC, 1995), histopathological diagnosis should be comparable over the study period.

In summary, we identified a decreasing trend in the intestinal type in younger age groups, and a stable trend in the diffuse type in the Lauren classification. In the JRSGC classification, three trend patterns were identified; a decreasing trend pattern for most age groups, a decreasing trend pattern only for younger age groups, and a stable trend pattern for the most age groups. Considering the changes in lifestyles of the Japanese during the recent three or four decades, our results suggest that there exist three types of gastric cancer from an aetiological and epidemiological point of view.

### ACKNOWLEDGEMENTS

This work was supported in part by a Grand-in-Aid for International Scientific Research (Special Cancer Research: 09042013) from the Ministry of Education, Science and Culture, and a Grant for International Health Cooperation Research (10C-4) from the Ministry of Health and Welfare, Japan.

### REFERENCES

Abe Y, Onodera M, Kamiya K, Saito F and Matsue T (1991) On the revision of the age-adjusted death rate. In: *Kousei no Shihyo*, pp 12–16. Health and Welfare Statistics Association: Tokyo (in Japanese)

Amorosi A, Bianchi S, Buiatti E, Cipriani F, Palli D and Zampi G (1988) Gastric cancer in a high-risk area in Italy. Histopathologic patterns according to Lauren's classification. *Cancer* **62**: 2191–2196

Asaka M, Kimura T and Kato M (1992) Relationship of *Helicobacter pylori* to serum pepsinogens in an asymptomatic Japanese population. *Gastroenterology* **102**: 760–766

Asaka M, Kimura T, Kato M, Kudo M, Miki K, Ogoshi K, Kato T, Tatsuta M and Graham DY (1994) Possible role of Helicobacter pylori infection in early gastric cancer development. *Cancer* **73**: 2691–2694

Crespi M and Citarda F (1998) *Helicobacter pylori* and gastric cancer: what is the real risk? *Gastroenterologist* **6**: 16–20

Eslick GD, Lim LL, Byles JE, Xia HH and Talley NJ (1999) Association of Helicobacter pylori infection with gastric carcinoma: a meta-analysis. *Am J Gastroenterol* **94**: 2373–2379

Hanai A and Fujimoto I (1982) Cancer incidence in Japan in 1975 and changes of epidemiological features for cancer in Osaka. *Natl Cancer Inst Monogr* **62**: 3–7

Hansson LE, Bergstrom R, Sparen P and Adami HO (1991) The decline in the incidence of stomach cancer in Sweden 1960–1984: a birth cohort phenomenon. *Int J Cancer* **47**: 499–503

Haruma K, Okamoto S, Kawaguchi H, Gotoh T, Kamada T, Yoshihara M, Sumii K and Kajiyama G (1997) Reduced incidence of Helicobacter pylori infection in young Japanese persons between the 1970s and the 1990s. *J Clin Gastroenterol* **25**: 583–586

Hirayama T (1984) Epidemiology of stomach cancer in Japan with special reference to the strategy for the primary prevention. *Jpn J Clin Oncol* **14**: 159–168

Howson CP, Hiyama T and Wynder EL (1986) The decline in gastric cancer: epidemiology of an unplanned triumph. *Epidemiol Rev* **8**: 1–27

Japanese Research Society for Gastric Cancer (1995) *Japanese Classification of Gastric Carcinoma*. Kanehara & Co., Ltd: Tokyo

Lauren P (1965) The two histological main types of gastric carcinoma: diffuse and so-called intestinal type carcinoma. *Acta Path Microbiol Scand* **64**: 31–49

Lauren PA and Nevalainen TJ (1993) Epidemiology of intestinal and diffuse types of gastric carcinoma. A time-trend study in Finland with comparison between studies from high- and low-risk areas. *Cancer* **71**: 2926–2933

Munoz N and Connelly R (1971) Time trends of intestinal and diffuse types of gastric cancer in the United States. *Int J Cancer* **8**: 158–164

Munoz N, Correa P, Cuello C and Duque E (1968) Histologic types of gastric carcinoma in high- and low-risk areas. *Int J Cancer* **3**: 809–818

National Cancer Center. (1972) *Zenkoku Igan Touroku*, Vol. 1. National Cancer Center: Tokyo (in Japanese)

Nikulasson S, Hallgrimsson J, Tulinius H, Sigvaldason H and Olafsdottir G (1992) Tumours in Iceland. 16. Malignant tumours of the stomach. Histological classification and description of epidemiological changes in a high-risk population during 30 years. *Apmis* **100**: 930–941

Nomura A, Stemmermann GN, Chyou PH, Kato I, Perez Perez GI and Blaser MJ (1991) *Helicobacter pylori* infection and gastric carcinoma among Japanese Americans in Hawaii. *N Engl J Med* **325**: 1132–1136

Oota K and Sobin LH (1977) *Histological typing of gastric and oesophageal tumors.* International Histological Classification of Tumors. World Health Organization: Geneva

Parsonnet J, Friedman GD, Vandersteen DP, Chang Y, Vogelman JH, Orentreich N and Sibley RK (1991a) *Helicobacter pylori* infection and the risk of gastric carcinoma. *N Engl J Med* **325**: 1127–1131

Parsonnet J, Vandersteen D, Goates J, Sibley RK, Pritikin J and Chang Y (1991b) *Helicobacter pylori* infection in intestinal- and diffuse-type gastric adenocarcinomas. *J Natl Cancer Inst* **83**: 640–643

Research Group for Population-based Cancer Registration in Japan (1999a) Cancer incidence and incidence rates in Japan in 1994: estimates based on data from seven population-based cancer registries. *Jpn J Clin Oncol* **29**: 361–364

Research Group for Population-based Cancer Registration in Japan (1999b) Cancer Incidence in Japan. In: *Monograph on Cancer Research No. 47*,

 *© 2001 Cancer Research Campaign*

Tominaga S and Oshima A (eds), pp 83–143. Japan Scientific Societies Press: Tokyo

Sipponen P, Kosunen TU, Valle J, Riihela M and Seppala K (1992) *Helicobacter pylori* infection and chronic gastritis in gastric cancer. *J Clin Pathol* **45**: 319–323

STATA Corp (1999) Glogit – Logit and probit on grouped data. In: *STATA Reference Manual Release 6*, pp 537–543. Stata Press: Texas

Talley NJ, Zinsmeister AR, Weaver A, DiMagno EP, Carpenter HA, Perez Perez GI and Blaser MJ (1991) Gastric adenocarcinoma and *Helicobacter pylori* infection. *J Natl Cancer Inst* **83**: 1734–1739

Tominaga S (1987) Decreasing trend of stomach cancer in Japan. *Jpn J Cancer Res* **78**: 1–10

Tominaga S and Kuroishi T (1997) An ecological study on diet/nutrition and cancer in Japan. *Int J Cancer* **Suppl 10**: 2–6

*© 2001 Cancer Research Campaign*
